# Immunoinflammatory biomarkers as predictors of hemorrhagic transformation in acute ischemic stroke patients after endovascular thrombectomy

**DOI:** 10.3389/fneur.2025.1606563

**Published:** 2025-06-13

**Authors:** Li Bao, Yuhang Wang, Shuang He

**Affiliations:** Department of Stroke Center, Affiliated Hospital of Nantong University, Nantong, China

**Keywords:** hemorrhagic transformation (HT), acute ischemic stroke (AIS), endovascular thrombectomy (EVT), symptomatic intracranial hemorrhage (sICH), IL-6, neutrophil-to-albumin ratio, immunoinflammatory biomarkers

## Abstract

**Background:**

Hemorrhagic transformation (HT) and symptomatic intracranial hemorrhage (sICH) are common complications of endovascular thrombectomy (EVT) in acute ischemic stroke (AIS) patients. The role of peripheral immune inflammation in HT after EVT is unclear. This study aimed to evaluate the relationship between immune inflammatory factor levels and HT and sICH occurrence, and to develop predictive models.

**Methods:**

We included 81 AIS patients who underwent EVT. Peripheral blood samples were collected immediately post-EVT to measure immunoinflammatory markers. Least absolute shrinkage and selection operator (LASSO) regression was used to select variables, and backward stepwise multivariable logistic regression identified independent predictors and predictive models for HT and sICH. The models’ discrimination was assessed using the area under the receiver operating characteristic curve (AUC), and calibration was evaluated using the Hosmer–Lemeshow test. Logistic regression models were used to evaluate the impact of HT or sICH on 90-day functional outcomes and mortality.

**Results:**

The HT rate was 39.51% (32/81), and the sICH rate was 17.07% (14/81). Multivariate analysis revealed that HT after EVT was significantly associated with collateral score [OR 0.27 (95% CI 0.13–0.52), *p* < 0.001], arteriosclerosis etiology [OR 0.11 (95% CI 0.02–0.46), *p* = 0.006], puncture to recanalization time [OR 3.72 (95% CI 1.07–14.59), *p* = 0.04], and levels of IL-6 [OR 7.33 (95% CI 2.1–31.07), *p* = 0.003; AUC 0.696 (95% CI 0.593–0.799)]. sICH was independently related to direct aspiration (DA) techniques [OR 0.07 (95% CI 0.09–0.35), *p* = 0.004] and neutrophil-to-albumin ratio (NAR) values [OR 5.69 (95% CI 1.16–37.24), *p* = 0.044; 0.676 (95% CI 0.550–0.803)]. Both predictive models for HT [AUC 0.898 (95% CI 0.831–0.965)] and sICH [AUC 0.925 (0.853–0.997)] exhibited good discrimination and calibration.

**Conclusion:**

IL-6 and NAR are potential biomarkers for predicting HT and sICH in AIS patients after EVT. This study developed simple and effective predictive models for HT and sICH based on immunoinflammatory factors. Future research should explore the spatiotemporal effects of immune inflammation on prognosis in AIS patients undergoing EVT.

## Introduction

Acute ischemic stroke (AIS) is characterized by high incidence, disability, and mortality rates, thereby imposing a significant burden on healthcare systems globally (1, 2). Endovascular thrombectomy (EVT) has emerged as an effective and widely adopted reperfusion therapy for AIS due to large vessel occlusion (LVO) (3–5). Nonetheless, the common and serious complication of EVT, hemorrhagic transformation (HT), is strongly correlated with poor clinical outcomes and can substantially undermine the therapeutic advantages of EVT (6, 7). Consequently, HT is frequently employed as a safety outcome in clinical research. Therefore, identifying risk factors for HT is crucial for optimizing clinical decision-making and enhancing postoperative management strategies.

Recent studies have identified several risk factors for HT following EVT, including the National Institutes of Health Stroke Scale (NIHSS) score, admission blood glucose levels, hyperdense middle cerebral artery sign, and symptom onset to puncture time (8–11). Furthermore, HT f post-EVT is strongly linked to blood-brain barrier (BBB) disruption, which is induced by reperfusion-related inflammation (12). Reperfusion increases reactive oxygen species (ROS) generation and heightens BBB permeability, facilitating the recruitment and activation of resident brain immune cells, such as microglia and astrocytes, as well as infiltrating blood-derived immune cells, including neutrophils and lymphocytes (13, 14). These immune cells release a diverse array of immunoinflammatory cytokines that modulate the balance between pro-inflammatory and anti-inflammatory responses, which is essential for maintaining BBB integrity (15, 16). However, there is a paucity of evidence regarding the relationship between peripheral blood immunoinflammatory cytokine levels and HT following EVT. This prospective study aimed to investigate the impact of immediate post-EVT peripheral blood immunoinflammatory cytokine levels on HT and symptomatic intracranial hemorrhage (sICH) in AIS patients, with the goal of facilitating early prediction of HT and sICH, thereby guiding treatment strategies.

## Methods

### Study design and management

From January 2024 to March 2025, consecutive adult AIS patients who underwent EVT for large vessel occlusion (LVO) at our center were recruited. The exclusion criteria included: (1) absence of post-EVT non-contrast computed tomography (NCCT) data of the head; (2) lack of immediate post-EVT intravenous blood samples; (3) patients with active infection, or those who had received antibiotics or immunosuppressive therapy within 2 weeks prior to stroke; (4) NCCT evidence of intracranial hemorrhage (ICH) before EVT; (5) procedure-related ICH, such as vascular perforation or arterial dissection; (6) patients with brain tumors, intracranial aneurysms, vascular malformations, or other intracranial space-occupying lesions; (7) patients with coagulopathy, hemorrhagic diseases, or a history of malignancy. All inclusion and exclusion criteria were determined by two trained senior neurologists. This study was approved by the Institutional Review Board (2021-Q094-02), and written informed consent was obtained from all enrolled patients.

All included patients underwent EVT under general anesthesia following the current AIS treatment guidelines, performed by experienced neurointerventionists. The selection of endovascular device [e.g., long sheath (NeuronMax, Penumbra, United States; Ballast, Balt, France; Gmax, Genesis, China), aspiration catheter (Ace, Penumbra, United States; React, Medtronic, United States), distal access catheter (Zenith, China; Soft, TONBRIDGE, China; Easyport, YIJIE, China; Navien, EV3, United States; Wellthrough, INT MEDICAL, China), microcatheter (Rebar, Medtronic, United States; Prowler Select plus, Codma, United States), microwire (Synchro SELECT, Stryker, United States; Avigo, Medtronic, United States; Traxcess, MicroVention, United States), and stents (Solitaire; Medtronic, United States; EmboTrap, Cerenovus, United States)], changes in thrombectomy strategy or device, and decisions regarding any salvage procedures (e.g., intracranial angioplasty with or without stenting and intra-arterial drug therapy), were determined by the operator, considering the pathogenesis of occlusion, the patient’s clinical condition, and other relevant factors.

Post-procedure, patients were closely monitored and managed in the stroke center. NCCT was performed 24–36 h post-EVT or promptly if neurological deterioration occurred, to exclude HT. Imaging assessments were conducted by experienced neurologists and radiologists blinded to clinical information. In cases of HT on NCCT, repeat NCCT was performed to assess the hemorrhage expansion or absortion, and to differentiate HT from contrast leakage. Systolic blood pressure was controlled below 140 mmHg in patients with successful reperfusion and below 160 mmHg in others.

### Data collection and outcomes

We collected demographic characteristics, medical history, pre-stroke modified Rankin Scale (mRS) score, and stroke characteristics, including the NIHSS score at admission, stroke etiology by TOAST criteria (17), blood glucose level at admission, systolic blood pressure at admission, Alberta Stroke Program Early CT Score (ASPECTS) at admission, the occlusion site, and collateral circulation score based on the American Society of Interventional and Therapeutic Neuroradiology/Society of Interventional Radiology (ASITN/SIR) classification (18). Treatment details included symptom onset to puncture time, the modified thrombolysis in cerebral infarction (mTICI) score at the final intracranial angiogram, puncture to recanalization time, the use of intravenous tissue plasminogen activator (IV tPA), EVT technique [direct aspiration (DA), stent retriever (SR), or combined DA and SR], and the number of passes.

Venous blood samples required for laboratory tests (including complete blood counts, biochemical markers, blood lipids, and immunoinflammatory cytokines) were collected immediately after the final angiography according to standard institutional guidelines. The neutrophil-to-lymphocyte ratio (NLR), systemic immune-inflammation index (SII), platelet-to-lymphocyte ratio (PLR), lymphocyte-to-monocyte ratio (LMR), and neutrophil-to-albumin ratio (NAR) were defined as described in previous studies (19–21). A Navios flow cytometer (Beckman Coulter, California, United States) was used to measure the plasma levels of IL-1β, IL-2, IL-4, IL-5, IL-6, IL-8, IL-10, IL-12p70, IL-17, IFN-γ, IFN-α, and TNF-α.

Safety outcomes were HT indicated by NCCT performed 24–36 h after EVT and sICH. HT and sICH were identified and classified according to the ECASS II (European Cooperative Acute Stroke Study) classification (22): HI1 was defined as small petechiae along the margins of the infarct; HI2 as confluent petechiae within the infarcted area without space-occupying effect; PH1 as blood clots in 30% of the infarcted area with slight space-occupying effect; and PH2 as blood clots ≥30% of the infarcted area with substantial space-occupying effect. Any ICH visible on NCCT with neurological deterioration (NIHSS score increase ≥4 points) was defined as sICH. Functional outcomes were the 90-day good outcome, defined as mRS scores of 0–2.

### Statistical analysis

Statistical analyses were conducted using R Studio (version 4.4.2). The Shapiro–Wilk test was applied to assess the normality of continuous variables. Normally distributed continuous variables were presented as mean ± standard deviation (SD); non-normally distributed continuous variables were presented as median (IQR); categorical variables were presented as frequency (percentage). Univariate comparisons for continuous variables were conducted using the independent *t*-test for normally distributed data and the Mann–Whitney *U* test for non-normally distributed data. For categorical variables, differences were assessed using the chi-squared test or Fisher’s exact test, depending on the expected cell counts. In multivariable analyses, continuous variables were dichotomized based on their median values to simplify interpretation. To address multicollinearity and overfitting, variable selection was performed using least absolute shrinkage and selection operator (LASSO) regression (23). The optimal penalty parameter (*λ*) was determined using *λ*_min_ + 1SE approach. Variables with non-zero coefficients from the LASSO regression were then included in a backward stepwise logistic regression based on the Akaike information criterion (AIC) to identify the best-fitting predictive models and independent predictors for HT and sICH. The discriminative performance of the selected models and independent predictors was assessed using the area under the receiver operating characteristic (ROC) curve (AUC). Model calibration was assessed using the Hosmer–Lemeshow test. A two-sided *p*-value of <0.05 was considered statistically significant.

## Results

**Figure 1:**
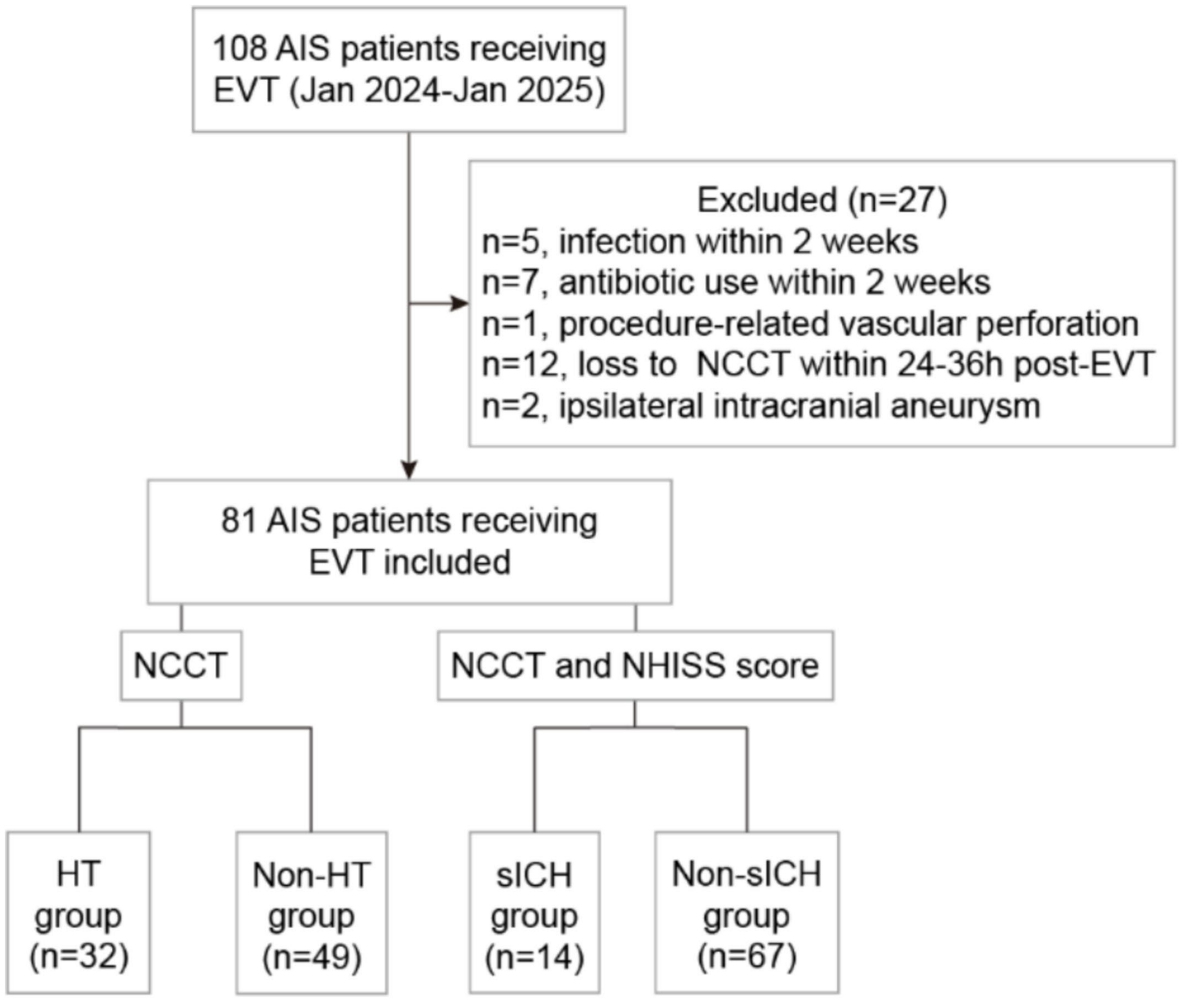
Study flowchart. AIS, acute ischemic stroke; EVT, endovascular thrombectomy; NCCT, non-contrast computed tomography; NHISS, National Institutes of Health Stroke Scale; HT, hemorrhagic transformation; sICH, symptomatic intracranial hemorrhage.

A total of 108 AIS patients receiving EVT were enrolled for this study. Five patients were excluded due to infection within 2 weeks prior to the stroke; seven patients were excluded for receiving antibiotics within 2 weeks prior to the stroke; one patient was excluded due to procedure-related ICH caused by vascular perforation; 12 patients were excluded due to the absence of NCCT within 24–36 h post-EVT; and two patients were excluded due to the ipsilateral intracranial aneurysms. Ultimately, 81 patients were included in this study. Complete data were successfully obtained for venous blood samples immediately following EVT, along with clinical characteristics, imaging and follow-up outcomes (Figure 1).

In the overall cohort, the median age was 72 years (range 65–76), with males comprising 62.96% (51/81). The rate of reperfusion (mTICI ≥2b) was 91.36% (74/81). The percentage of HT patients was 39.51% (32/81), including 3 HI1, 8 HI2, 13 PH1 and 8 PH2. The incidence of sICH was 17.07% (14/81). Among patients receiving IV-tPA combined with EVT, the rates of HT and sHT were 47.83% (11/23) and 30.43% (7/23), respectively; whereas in patients receiving EVT alone, the rates of HT and sHT were 36.21% (21/58) and 12.07% (7/58), respectively. At the 90-day follow-up, 45 patients (55.56%) achieved a good functional outcome (mRS 0–2). The 90-day mortality rate was 12.35% (10/81). Patients were divided into two groups: the HT group (*n* = 32) and non-HT group (*n* = 49), or the sICH group (*n* = 14) and non-sICH group (*n* = 67). Clinical characteristics, treatment details, functional outcomes, and univariate analysis results were presented in Table 1.

**Table 1:** Univariate analysis in clinical characteristics, treatment details and functional outcomes.

Variables	HT (*n* = 32)	Non-HT (*n* = 49)	*p*	sICH (*n* = 14)	Non-sICH (*n* = 67)	*p*
Clinical characteristics						
Age (years), median (IQR)	73.5 (69–76.75)	71 (62–75.5)	0.09	74 (68.25–77.25)	71 (64–76)	0.34
Female	14 (43.75%)	16 (32.65%)	0.35	6 (42.86%)	24 (35.82%)	
BMI (kg/m^2^), median (IQR)	25.55 (22.49–27.34)	24.16 (20.73–26.61)	0.1	26.04 (22.64–27.83)	24.16 (20.76–26.89)	0.12
Hypertension	26 (81.25%)	37 (75.51%)	0.6	11 (78.57%)	52 (67.53%)	0.54
Diabetes	10 (31.25%)	18 (36.73%)	0.64	5 (35.71%)	23 (34.33%)	>0.99
Atrial fibrillation	17 (53.13%)	15 (30.61%)	0.06	6 (42.86%)	26 (38.81%)	0.77
Coronary heart disease	13 (40.63%)	8 (16.33%)	0.02^a^	2 (14.29%)	19 (28.36%)	0.34
Dyslipidemia	18 (56.25%)	17 (34.69%)	0.07	9 (64.29%)	26 (38.81%)	0.14
Smoking	15 (46.88%)	10 (20.41%)	0.02^a^	7 (50.00%)	18 (26.87%)	0.11
Alcohol abuse	13 (40.63%)	19 (38.78%)	>0.99	7 (50.00%)	25 (37.31%)	0.39
History of antiplatelet drugs	4 (12.5%)	8 (16.33%)	0.76	2 (14.29%)	10 (14.93%)	>0.99
Previous stroke	6 (18.75%)	8 (16.33%)	0.77	1 (7.14%)	18 (26.87%)	0.17
Pre-stroke mRS, median (IQR)	0 (0–1)	0 (0–1)	0.81	0 (0–1)	0 (0–1)	0.51
Etiology by TOAST			0.002^a^			0.01^a^
Atherosclerosis	3 (9.38%)	22 (44.9%)		0	25 (37.31%)	
Cardioembolism	18 (56.25%)	12 (24.49%)		7 (50%)	23 (34.33%)	
Other reason	8 (25%)	12 (24.49%)		5 (35.71%)	15 (22.39%)	
Undetermined	3 (9.38%)	3 (6.12%)		2 (14.29%)	4 (5.97%)	
Occlusion site			0.46			0.44
ICA	3 (9.38%)	10 (20.41%)		2 (14.29%)	11 (16.42%)	
MCA	26 (81.25%)	32 (65.31%)		9 (64.29%)	49 (73.13%)	
ACA	0	1 (2.04%)		0	1 (1.49%)	
VA	0	2 (4.08%)		0	2 (2.99%)	
BA	3 (9.38%)	4 (8.16%)		3 (21.43%)	4 (5.97%)	
Glucose level at admission (mmol/L), median (IQR)	8.1 (6–11.35)	6.2 (5.7–9.3)	0.15	8.45 (6.08–11.95)	6.4 (5.7–9.4)	0.15
Systolic blood pressure at admission (mm Hg), median (IQR)	156.1 ± 25.94	148.6 ± 18.35	0.16	164.8 ± 27.22	148.8 ± 19.67	0.01^a^
NIHSS score at admission, median (IQR)	19 (13–21.75)	12 (10–17.5)	<0.001^a^	18.5 (12–21.25)	14 (10–19)	0.13
ASPECTS at admission	10 (8–10)	9 (8–10)	0.61	10 (8–10)	9 (8–10)	0.73
Collateral score	1 (0.25–2)	2 (1–2)	0.004^a^	1 (0.75–1)	2 (1–2)	0.02^a^
Treatment details						
IV tPA	11 (34.38%)	12 (24.49%)	0.45	7 (50%)	16 (23.88%)	0.1
Symptom onset to puncture time (min), median (IQR)	325.5 (286.3–467.8)	383 (291–570)	0.18	320 (271.3–408.8)	390 (290–560)	0.12
Puncture to recanalization time (min)	96 (62–119.5)	81 (63–103)	0.1	115 (70.25–172.5)	82 (62–106)	0.01^a^
EVT technique			0.91			0.01^a^
DA	14 (43.75%)	23 (46.94%)		2 (14.29%)	35 (52.24%)	
SR	6 (18.75%)	10 (20.41%)		3 (21.43%)	13 (19.4%)	
DA + SR	12 (37.5%)	16 (32.65%)		9 (64.29%)	19 (28.36%)	
No. of passes	2.5 (2–4)	2 (1–3)	0.03^a^	4 (2–4)	2 (1–3)	0.001^a^
mTICI score at final intracranial angiogram						
≥2b	29 (90.63%)	45 (91.84%)	>0.99	11 (78.57%)	63 (94.03%)	0.1
3	19 (59.38%)	32 (65.31%)	0.64	8 (57.14%)	43 (64.18%)	0.76
Immediate laboratory examination data after EVT						
Neutrophils (×10^9^/L)	8.86 (5.77–11.69)	7.54 (5.88–8.76)	0.26	9.66 (7.09–14.52)	7.29 (5.8–8.94)	0.008^a^
Lymphocytes (×10^9^/L)	0.91 (0.60–1.19)	1.22 (0.90–1.56)	0.01^a^	0.93 (0.57–1.19)	1.12 (0.78–1.45)	0.14
Monocytes (×10^9^/L)	0.53 (0.3–0.76)	0.49 (0.39–0.66)	0.99	0.65 (0.25–0.82)	0.49 (0.37–0.66)	0.63
Hb (g/L)	131 (118.5–140.3)	132 (116.5–143.5)	0.55	133.5 (123.5–146.3)	131 (114–142)	0.3
PLT (×10^9^/L)	177.1 ± 61.14	193.3 ± 54.53	0.22	198.1 ± 67.84	184.5 ± 55.28	0.43
ALB (g/L)	35.73 ± 5.1	36.73 ± 5.49	0.41	37.48 ± 3.72	36.09 ± 5.6	0.38
TC (mmol/L)	4.3 ± 1.24	4.68 ± 1.12	0.16	4.58 ± 1.3	4.52 ± 1.16	0.87
LDL (mmol/L)	2.84 ± 0.92	3.13 ± 0.83	0.14	3.03 ± 0.91	3.01 ± 0.87	0.96
NLR	10.47 (5.7–17.8)	5.83 (4.7–10.5)	0.02^a^	11.91 (8.23–20.41)	6.28 (4.61–11.68)	0.03^a^
SII	1730 (1278–2,642)	1,303 (806.3–1929)	0.05	1816 (1376–3,746)	1,358 (885.9–2,226)	0.02^a^
PLR	201.1 (159.7–272.5)	163.5 (122.9–210)	0.04^a^	219.7 (157.7–284.4)	169.7 (124.2–240.8)	0.1
LMR	16.29 (10.68–22.74)	14.34 (10.79–18.95)	0.3	19.1 (10.9–53.05)	14.48 (10.73–19.02)	0.22
NAR	0.23 (0.18–0.3)	0.2 (0.17–0.25)	0.11	0.28 (0.2–0.4)	0.2 (0.17–0.25)	0.02^a^
IL1β (pg/mL)	0.17 (0.14–0.28)	0.15 (0.09–0.24)	0.29	0.18 (0.16–0.34)	0.16 (0.09–0.23)	0.047^a^
IL2 (pg/mL)	0.43 (0.07–1.04)	0.31 (0.08–0.74)	0.31	0.75 (0.27–1.08)	0.31 (0.06–0.74)	0.1
IL4 (pg/mL)	1.51 (1.04–2.34)	1.3 (0.85–1.79)	0.26	1.92 (1.32–3.24)	1.32 (0.8–1.81)	0.01^a^
IL5 (pg/mL)	1.17 (0.65–1.53)	1.05 (0.66–1.51)	0.86	1.19 (1.02–1.59)	1.05 (0.61–1.55)	0.34
IL6 (pg/mL)	10.79 (5.04–19.4)	5.5 (3.5–7.06)	0.002^a^	8.53 (4.4–18.62)	5.98 (4.08–11.28)	0.58
IL8 (pg/mL)	12.17 (6.25–25.31)	9.19 (4.53–17.98)	0.18	9.73 (6.18–17.55)	9.47 (4.99–23.25)	0.93
IL10 (pg/mL)	4.15 (2.09–6.21)	2.88 (2.11–4.24)	0.26	5.05 (2.44–6.61)	2.88 (1.93–4.51)	0.05
IL12p70 (pg/mL)	0.06 (0.04–0.89)	0.06 (0.04–0.31)	0.54	0.06 (0.04–1.12)	0.06 (0.04–0.36)	0.46
IL17 (pg/mL)	0.35 (0.23–0.62)	0.34 (0.19–0.53)	0.56	0.39 (0.32–0.74)	0.32 (0.15–0.53)	0.15
IFNα (pg/mL)	0.47 (0.26–0.87)	0.43 (0.28–0.68)	0.98	0.49 (0.39–0.95)	0.43 (0.26–0.68)	0.14
IFNγ (pg/mL)	1.94 (1.12–3.06)	1.78 (1.11–2.59)	0.5	2.77 (1.89–3.54)	1.62 (1.03–2.59)	0.02^a^
TNFα (pg/mL)	1.71 (1.27–2.75)	1.68 (1.25–2.43)	0.7	1.9 (1.37–2.96)	1.68 (1.25–2.47)	0.46
90-day good outcome	11 (34.38%)	34 (69.39%)	0.003^a^	4 (28.57%)	41 (61.19%)	0.04^a^
90-day mortality	8 (25%)	2 (4.08%)	0.01^a^	6 (42.86%)	4 (5.97%)	0.001^a^

HT, hemorrhagic transformation; sICH, symptomatic intracranial hemorrhage; BMI, body mass index; mRS, modified Rankin Scale; ICA, internal carotid artery; MCA, middle carotid artery; ACA, anterior cerebral artery; VA, vertebral artery; BA, basilar artery; NHISS, National Institutes of Health Stroke Scale; ASPECTS, Alberta Stroke Program Early CT Score; IV tPA, intravenous tissue plasminogen activator; EVT, endovascular thrombectomy; mTICI, modified thrombolysis in cerebral infarction; Hb, hemoglobin; PLT, platelet; ALB, albumin; TC, triglyceride; LDL, low density lipoprotein; NLR, neutrophil-to-lymphocyte ratio; SII, systemic immune-inflammation index; PLR, platelet-to-lymphocyte ratio; LMR, lymphocyte-to-monocyte ratio; NAR, neutrophil-to-albumin ratio; IL, interleukin; IFN, interferon; TNF, tumor necrosis factor.

The inter-group comparison was performed using the independent *t*-test or the Mann–Whitney *U* test for continuous variables, and the chi-squared test or Fisher’s exact test for categorical variables.

a *p*-value less than 0.05.

### Univariate analysis

Compared with the non-HT group, the HT group exhibited a significantly higher proportion of patients with coronary artery disease (40.63% vs. 16.33%, *p* = 0.02) and smoking patients (46.88% vs. 20.41%, *p* = 0.02). The stroke etiology analysis revealed significant differences between the HT group, sICH group, and the negative control group (*p* = 0.002). In the positive groups, cardioembolism was the predominant etiology (56.25% for HT group and 50% for sICH group), whereas atherosclerosis was the primary cause in the negative control group (44.9%). The NHISS score at admission was significantly higher in the HT group compared to the non-HT group [19 (13–21.75) vs. 12 (10–17.5), *p* < 0.001]. sICH patients had significantly higher mean systolic blood pressure at admission (164.8 ± 27.22 vs. 148.8 ± 19.67, *p* = 0.01). The occurrence of HT or sICH after EVT was significantly associated with worse collateral circulation (*p* = 0.004 for HT and *p* = 0.02 for sICH). No other differences were observed in demographic, clinical, or stroke characteristics between the groups.

In terms of treatment details, the number of passes attempting for thrombectomy was significantly associated with the occurrence of HT and sICH [2.5 (2–4) vs. 2 (1–3), *p* = 0.03 for HT; 4 (2–4) vs. 2 (1–3), *p* = 0.001 for sICH]. Furthermore, the puncture to recanalization time in the sICH group was significantly longer than in the non-sICH group [115 (70.25–172.55) vs. 82 (62–106), *p* = 0.01]. Notably, EVT technique also differed significantly between the sICH and non-sICH groups (*p* = 0.01), with DA + SR being the predominant technique in sICH patients (64.29%) and DA being more common in non-sICH patients (52.24%).

In laboratory findings, we observed that HT patients had lower lymphocyte counts (*p* = 0.01), higher NLR values (*p* = 0.02), and higher IL-6 levels (*p* = 0.002) in immediate post-EVT venous blood samples; sICH patients exhibited higher neutrophil counts (*p* = 0.008), elevated values of NLR (*p* = 0.03), SII (*p* = 0.02) and NAR (*p* = 0.02), increased levels of IL-1β (*p* = 0.047), IL-4 (*p* = 0.01), and IFN-γ (*p* = 0.02).

Compared to the non-HT group, the HT group demonstrated a significantly lower rate of 90-day good functional outcomes (34.38% vs. 69.39%, *p* = 0.003) and a higher mortality rate (25% vs. 4.08%, p = 0.01). Similarly, sICH was significantly associated with worse 90-day functional outcomes (*p* = 0.04) and higher 90-day mortality (*p* = 0.001).

### Variable selection, multivariate regression analysis and model performance evaluation

**Figure 2:**
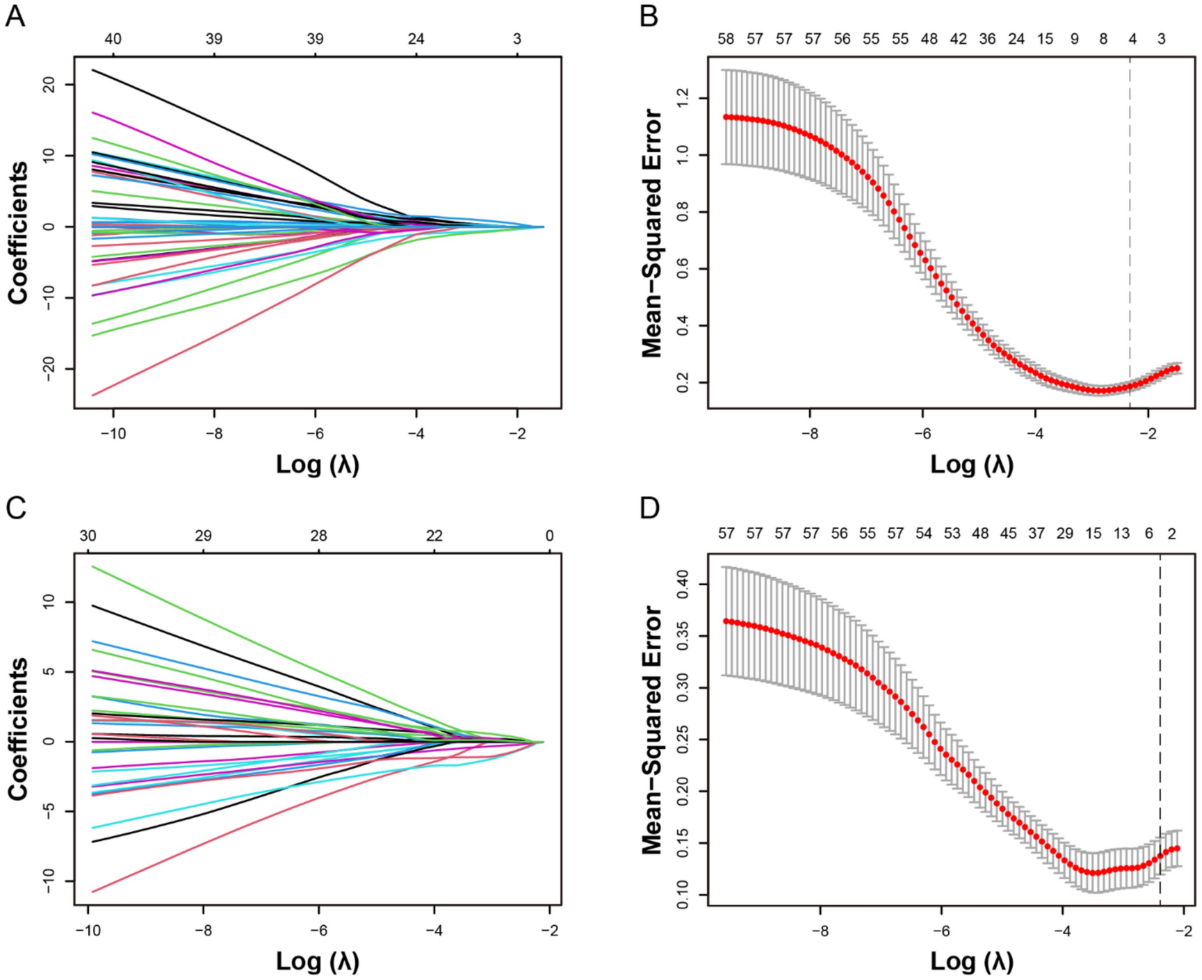
Selection of potential predictive factors for hemorrhagic transformation (HT) and symptomatic intracranial hemorrhage (sICH) using the least absolute shrinkage and selection operator (LASSO) regression. The LASSO coefficient plots for the dependent variables HT **(A)** and sICH **(C)** were shown. The 10-fold cross-validation for parameter selection (*λ*) in the LASSO Logistic regression models for HT **(B)** and sICH **(D)** was presented. The optimal *λ* is selected as *λ*_min_ + 1SE, with *λ* = 0.0916 and log(*λ*) = −2.3226. The black vertical dashed line indicates the optimal value determined by the *λ*_min_ + 1SE criterion.

The optimal *λ* was selected using *λ*_min_ + 1SE criterion (*λ* = 0.0916). Using LASSO regression, five variables with non-zero coefficients associated with HT and six variables with non-zero coefficients associated with sICH were identified (Figure 2). These selected variables and the use of IV-tPA were then included in a backward stepwise logistic regression model to establish the optimal predictive model for HT and sICH, along with their independent predictive factors (Table 2). In Model A, which predicted HT (AIC = 73.06), all included variables were identified as independent predictors, including collateral score [OR 0.27 (95% CI 0.13–0.52), *p* < 0.001], arteriosclerosis etiology [OR 0.11 (95% CI 0.02–0.46), *p* = 0.006], puncture to recanalization time [OR 3.72 (95% CI 1.07–14.59), *p* = 0.04], and IL-6 [OR 7.33 (95% CI 2.1–31.07), *p* = 0.003]. In the Model B (AIC = 49.44) predicting sICH, DA [OR 0.07 (95% CI 0.09–0.35), *p* = 0.004] was identified as an independent protective factor for sICH, while NAR [OR 5.69 (95% CI 1.16–37.24), *p* = 0.044] was an independent risk factor.

**Table 2:** Multivariate logistic regression analysis.

Model A predicting HT			
Variables	*β*	OR (95%CI)	*p*
Collateral score	−1.29	0.27 (0.13–0.52)	<0.001^a^
Atherosclerosis etiology	−2.22	0.11 (0.02–0.46)	0.006^a^
Puncture to recanalization time	1.31	3.72 (1.07–14.59)	0.04^a^
IL6 (>5.98 pg/mL)	1.99	7.33 (2.1–31.07)	0.003^a^

Model B predicting sICH			
Variables	*β*	OR (95% CI)	*p*
Collateral score	−0.79	0.45 (0.18–1.01)	0.07
Atherosclerosis etiology	…	…	>0.99
DA	−2.66	0.07 (0.09–0.35)	0.004^a^
NAR (>0.205)	1.74	5.69 (1.16–37.24)	0.044^a^

HT, hemorrhagic transformation; sICH, symptomatic intracranial hemorrhage; IL, interleukin; NAR, neutrophil-to-albumin ratio.

a *p*-value less than 0.05.

**Figure 3:**
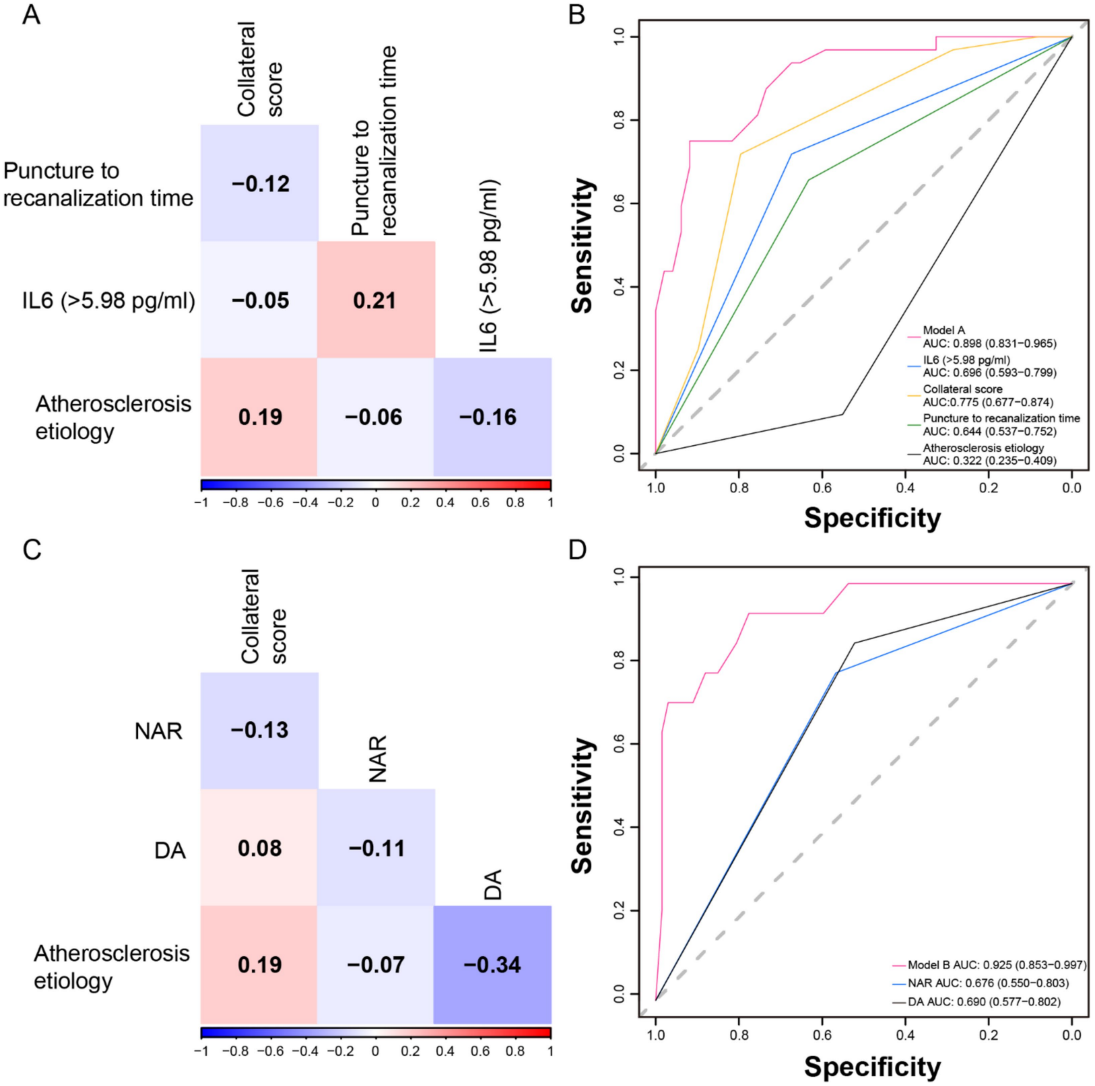
Multicollinearity test of variables, and receiver operating characteristic (ROC) curves of models and independent predictors. The correlation matrix indicated that there was no multicollinearity among the variables in Model A **(A)** and Model B **(C)**. **(B)** Model A and its independent predictor ROC curves. **(D)** Model B and its independent predictor ROC curves.

After confirming no multicollinearity among the variables (Figure 3A), the AUC for Model A in predicting HT was 0.898 (95% CI 0.831–0.965), indicating strong discriminatory performance (Figure 3B). The AUC value for IL-6 was 0.696 (95% CI 0.593–0.799). The Hosmer–Lemeshow test statistic was not significant (*χ*^2^ = 4.91, *p* = 0.84), indicating that Model A had good calibration. Similarly, after ensuring no multicollinearity among the variables in Model B (Figure 3C), the ROC curve demonstrated that Model B, which was associated with sICH, had excellent discrimination [AUC = 0.925 (0.853–0.997)], with the AUC for NAR alone being 0.676 (95% CI 0.550–0.803) (Figure 3D). Model B also exhibited good calibration (*χ*^2^ = 6.46, *p* = 0.69, as per the Hosmer–Lemeshow Test).

## Discussion

This study observed a post-EVT HT incidence of 39.51%, consistent with previous studies (9, 24, 25). The incidence of sICH was slightly higher (17.07%) compared to the large meta-analysis report (4.4%) (3), possibly due to the longer symptom onset to puncture time, higher proportion of hypertension, and higher admission blood glucose levels in our study cohort. Through LASSO and multivariate regression analysis, we found that the occurrence of HT within 24–36 h after EVT was independently associated with lower collateral score, non-atherosclerotic etiology, longer symptom to puncture time, and higher IL-6 levels. The occurrence of sICH was independently related to the use of non-DA thrombectomy techniques and higher NAR values. The predictive models for HT and sICH exhibited good discrimination and calibration. To our knowledge, this is the first study to explore the factors influencing the occurrence of HT and sICH after EVT incorporating peripheral immunoinflammatory cytokines.

EVT restored blood flow, which increased oxidative stress and promoted the release of inflammatory cytokines, triggering a cascade of pathological events. These events involved the activation of apoptotic pathways, BBB disruption, cerebral edema, and HT. Ultimately, ischemia–reperfusion injury exacerbated brain damage and neurological deficits (16, 26). IL-6 activates and recruits neutrophils and monocytes, stimulating endothelial cells to secrete adhesion molecules and other inflammatory mediators, thereby enhancing the local and general inflammatory response (27). Circulating and local IL-6 induces a pre-thrombotic state, stimulating the production of platelet-derived growth factor, fibroblast growth factor, and macrophage colony-stimulating factor, which in turn promotes smooth muscle cell proliferation (28). Large meta-analyses have demonstrated a dose–response relationship between peripheral IL-6 levels and the risk of ischemic stroke (29). Elevated peripheral IL-6 levels were significantly associated with lower first-pass effect rates (defined as achieving complete or near-complete reperfusion after first thrombectomy) (30) and futile EVT reperfusion (31). Peripheral serum IL-6 levels were considered a poor prognostic factor in AIS patients (32), correlating with imaging parameters such as the mean volume of diffusion weighted imaging lesions at admission, perfusion defects, and final infarct area (33). Recent studies indicated that IL-6 levels in intracranial ischemic arterial bed samples is associated with a lower 90-day mRS score (34). Our study found that IL-6 levels exceeding 5.98 pg/mL were significantly independently associated with HT after EVT. IL-6 alone demonstrated 69.6% accuracy in predicting HT, whereas the predictive model incorporating IL-6 achieved 89.8% accuracy. Therefore, IL-6 played a critical role throughout the AIS course, influencing onset, EVT efficiency, hemorrhagic complications, and functional recovery. Preclinical studies, however, suggested that IL-6 had a dual role, promoting neurogenesis, angiogenesis, and neuronal differentiation (35, 36). The uncertain neurotrophic effects of IL-6 may explain why IL-6 in our study predicted HT but not sICH (worsening neurological deficits). There are currently two registry clinical trials on cytokines in AIS patients (NCT05004389; NCT03297827). The value of the spatiotemporal distribution of IL-6 as an inflammatory marker in AIS deserves further investigation. Timely adjustment of high-risk factors for HT could be an effective measure to enhance post-EVT management, for example, the early use of IL-6 receptor inhibitors (37).

An increase in NAR reflected a worsening systemic inflammatory response, which might exacerbate brain edema and tissue damage by increasing BBB permeability. Albumin plays a key role in osmoregulation, antioxidation, and anti-inflammation (38). Low serum albumin levels are associated with an increased risk of ischemic stroke and ICH (39). High NAR values have been shown to be positively correlated with stroke severity (40), increasing the need for intensive care in AIS patients (41), and significantly associated with poor functional outcomes at 90 days (42). Recent studies have demonstrated that combining NAR with triglyceride levels into a noval index was significantly correlated with spontaneous HT in AIS patients (43). NAR has proven to be a promising biomarker value in prognostic studies of other cardiovascular and cerebrovascular diseases, such as subarachnoid hemorrhage and acute myocardial infarction without ST-segment elevation (44, 45). However, this study did not find a predictive effect of NAR on HT, possibly because the levels of peripheral neutrophils and albumin immediately after reperfusion had not yet reached their extreme values (46, 47). The primary contributors to HT, which promote neuroinflammation and disrupt the BBB, are the inflammatory products of multiple immune cells (such as IL-6). Neutrophils, as one of the “producers,” may have an insignificant role. The positive correlation between NAR and sICH (AUC = 0.676) suggested that in HT patients, once accompanied by neurological deterioration, the intense systemic immune-inflammatory response is closely associated with secondary brain cell injury. Therefore, in sICH patients with a strong neuroinflammatory response, the NAR levels in the early stage after reperfusion are often elevated, as can be observed from the intergroup comparison. This simple, cost-effective, and efficient inflammatory marker offers clinicians a dynamic, long-term tool to assess patient risk. For individualized postoperative management decisions in patients with high NAR levels, consideration can be given to the use of Cl-amidine liposomal nanocarriers (targeting the inhibition of central neutrophil extracellular traps), which have been shown to effectively reduce ischemic and reperfusion injury (48), as well as albumin-assisted therapy (49).

Consistent with previous studies on clinical and imaging predictors of HT (50–52), we also found that poorer collateral circulation scores and longer symptom onset to puncture time were significantly associated with HT. Both factors are related to prolonged BBB damage and the expansion of the core infarct. AIS patients with large artery atherosclerosis often present with well-developed collateral circulation. However, the pathological vascular calcification limits the initial reperfusion improvement, thereby attenuating reperfusion injury. Furthermore, we observed a negative correlation between DA as a first-line thrombectomy technique and the occurrence of sICH. A meta-analysis of 19 studies revealed that the combined DA + SR technique is associated with a higher risk of sICH (53). SR-induced endothelial injury may be a potential cause of increased blood-brain barrier permeability (54).

This study has several limitations. First, as a single-center study with a relatively small sample size, selection bias was inherent. Under the background of high heterogeneity of serum biomarkers, the retrospective nature of the study and the small sample size may impact the reliability and generalizability of the results. Second, although NCCT was used for dynamic evaluation of HT, the extravasation of contrast agents inevitably influenced the outcome observations. Moreover, the study population predominantly consisted of individuals with a high prevalence of large artery atherosclerosis, which limited the generalizability of the findings. Finally, dynamic assessment of peripheral immune inflammatory cytokine levels is essential for further research into their role, particularly at key time points such as admission, pre-procedure, and discharge.

## Conclusion

This study provided evidence that immunoinflammatory factor IL-6 in AIS patients after EVT was positively correlated with HT, and NAR was positively associated with sICH. Additionally, both HT and sICH were negatively correlated with prognosis. The study explored straightforward and effective biomarkers and predictive models for HT and sICH. These findings could provide valuable support for clinical decision-making and interventions designed to minimize the incidence of EVT-related complications such as HT and sICH, while offering new insights into optimizing treatment strategies for patients. Future research should further investigate the spatiotemporal dynamics of immune inflammation, its regulatory mechanisms, and advancements in EVT techniques to reduce the occurrence of these severe complications.

## Data Availability

The raw data supporting the conclusions of this article will be made available by the authors, without undue reservation.
